# MOTS-c repairs myocardial damage by inhibiting the CCN1/ERK1/2/EGR1 pathway in diabetic rats

**DOI:** 10.3389/fnut.2022.1060684

**Published:** 2023-01-04

**Authors:** Manda Wang, Gangqiang Wang, Xiaoli Pang, Jiacheng Ma, Jinghan Yuan, Yanrong Pan, Yu Fu, Ismail Laher, Shunchang Li

**Affiliations:** ^1^Institute of Sports Medicine and Health, Chengdu Sport University, Chengdu, China; ^2^Physical Education Section, Chengdu Textile College, Chengdu, China; ^3^Department of Pharmacology and Therapeutics, Faculty of Medicine, The University of British Columbia, Vancouver, BC, Canada

**Keywords:** MOTS-c, type 2 diabetes, mitochondrial, myocardium, transcriptome

## Abstract

Cardiac structure remodeling and dysfunction are common complications of diabetes, often leading to serious cardiovascular events. MOTS-c, a mitochondria-derived peptide, regulates metabolic homeostasis by accelerating glucose uptake and improving insulin sensitivity. Plasma levels of MOTS-c are decreased in patients with diabetes. MOTS-c can improve vascular endothelial function, making it a novel therapeutic target for the cardiovascular complications of diabetes. We investigated the effects of MOTS-c on cardiac structure and function and analyzed transcriptomic characteristics in diabetic rats. Our results indicate that treatment with MOTS-c for 8-week repaired myocardial mitochondrial damage and preserved cardiac systolic and diastolic function. Transcriptomic analysis revealed that MOTS-c altered 47 disease causing genes. Functional enrichment analysis indicated MOTS-c attenuated diabetic heart disease involved apoptosis, immunoregulation, angiogenesis and fatty acid metabolism. Moreover, MOTS-c reduced myocardial apoptosis by downregulating CCN1 genes and thereby inhibiting the activation of ERK1/2 and the expression of its downstream EGR1 gene. Our findings identify potential therapeutic targets for the treatment of T2D and diabetic cardiomyopathy.

## 1. Introduction

Diabetes mellitus (DM) is a metabolic disease characterized by persistent hyperglycemia caused by insulin resistance (IR) or insulin deficiency (1), and has a worldwide prevalence of nearly 300 million (2). Persistent hyperglycemia damages the structure and function of the myocardium in patients with DM, resulting in cardiac diastolic dysfunction that can lead to heart failure and myocardial infarction (3–6).

Mitochondrial derived peptides are small bioactive peptides produced by the short open reading frame region of mtDNA, but which does not possess the traditional characteristics of protein-coding genes (7). The mitochondrial open-reading-frame of the 12s rRNA-c (MOTS-c) inhibits the folate cycle, purine biosynthesis, and activates 5’AMP activated protein kinase (AMPK) (8). MOTS-c regulates the expression of nuclear genes related to metabolism, including the antioxidant response element (ARE) that protects against metabolic stress (9, 10). Serum levels of MOTS-c are reduced in patients with type 2 diabetes (11).

The functional activity of MOTS-c has not been widely studied, but in cell culture and animal models suggest that MOTS-c affects mitochondrial metabolism and insulin sensitivity (12), accelerates glucose uptake (8, 12) and regulates glucose production in liver (13, 14). Insulin-dependent protein kinase B (AKT) is activated by MOTS-c in mouse skeletal muscle cells (13). Treatment with MOTS-c activates the AMPK pathway and increases GLUT4 expression in skeletal muscle (8), suggesting that exogenous MOTS-c could lower hyperglycemic levels in patients with diabetes and thereby enhance cardiac function. Our preliminary study reported that exogenous MOTS-c increased the levels of endogenous MOTS-c protein expression in the myocardium (15). More importantly, our findings also demonstrated that MOTS-c prevented myocardial ultrastructural damage and improved cardiac function in non-diabetic rats (15).

We studied the effects of treatment with MOTS-c on cardiac structure and function of rats with diabetes by examining the myocardial transcriptome using the RNA-Seq technology. Our findings could help to identify novel potential therapeutic targets in the management of cardiac complications in diabetes.

## 2. Materials and methods

### 2.1. Animal care

Experiments were approved by the Academic Committee of Chengdu Sport Institute (No: 2021-07). Male Sprague Dawley (SD) rats (*n* = 40) were obtained from Chengdu Da-shuo Experimental Animals Co., Ltd. (Chengdu, China). All rats were housed in a pathogen-free animal room at Chengdu Sports University with relative humidity of 50∼60%, ambient temperatures of 21-23°C, and a 12-h dark/light cycle. The body weights (BW) of rats were measured once a week.

### 2.2. Induction of type 2 diabetes

Rats were randomly divided into control (C, *n* = 10) and pre-diabetic (PD, *n* = 30) groups. Rats in the PD group were fed a high-fat diet containing 67% normal pellets, 10% lard, 20% sucrose, 2% cholesterol, and 1% sodium cholate (16) for 7 weeks, and the insulin resistance index (HOMA-IRI) was determined for the rats. During the first 7 weeks of feeding with a high-fat diet, the pre-diabetic rats were given a single intraperitoneal injection of STZ (30 mg/kg) (Sigma-Aldrich, St Louis, MO) dissolved in sodium citrate buffer (0.1 mol/L, pH 4.4) (17, 18). Diabetes was confirmed when the blood glucose measurements were greater than 16.7 mmol/L (Table 1). Diabetic rats were randomly divided into two groups: (1) diabetes group (untreated) (D), (2) diabetic rats treated with MOTS-c (M).

**TABLE 1:** Timeline of interventions.

Timeline	Weeks 1-7	Week 8	Weeks 9-15
Interventions	High-fat diet (group PD) Normal diet (group C)	➀ Single intraperitoneal injection of STZ for group PD ➁ Test HOMA-IR and blood glucose to confirm diabetes	MOTS-c treatment for group M and saline for groups C and D

### 2.3. MOTS-c treatment protocol

MOTS-c is synthesized *in vitro* according to its amino acid sequence and spatial structure by GL Biochem (Shanghai) Ltd. (Chengdu, China). Diabetic rats in group M were injected with MOTS-c (0.5 mg/kg/day, i.p.) for 7 days/week for 8 weeks, while rats in groups C and D were injected with normal saline in the same way (Table 1).

### 2.4. Assessment of plasma glucose, insulin, and HOMA-IR indexes

Plasma glucose levels were estimated with a glucometer (On⋅Call, China). Insulin levels were measured with ELISA kits for rat insulin (Immuno Way, USA) with a plate reader (SpectraMax M5 from Thermo Scientific, USA) at 450 nm. The HOMA-IR was calculated as: HOMA-IR = Fasting blood glucose (FBG) (mmol/L) × Fasting insulin (FINS) (mU/L)/22.5 (19).

### 2.5. Measurement of cardiac structure and function

#### 2.5.1. Transmission electron microscopy (TEM)

Cardiac tissues were pre-fixed in 3% glutaraldehyde and post fixed in 1% osmium tetroxide, and then dehydrated in acetone and successively infiltrated with a dehydrating agent and epoxy resin at ratios of 3:1, 1:1, 1:3, and 30 to 60 min per step. The infiltrated samples were placed into molds, irrigated with embedding solution, then warmed and allowed to form polymerized embedding blocks. Ultrathin sections (∼50 nm thickness) were made using an ultramicrotome. After staining with uranyl acetate followed by lead citrate, images of cells were acquired using a JEM-1400PLUS TEM (JEOL, Japan).

#### 2.5.2. Echocardiography

Small animal echocardiography (Philips, CX50) was used to detect changes in ejection fraction (EF), left ventricular posterior wall diastole (LVPWd), early diastolic transmitral flow velocity (E wave), late (atrial) diastolic transmitral flow velocity (A wave) and early to late diastolic transmitral flow velocity (E/A). We measured the long axis image of the left ventricle and the short axis image of the middle part of the left ventricle by M-mode echocardiograms.

### 2.6. Construction of transcriptome

The cDNA library of samples was sequenced using sequencing by synthesis (SBS) technology with Illumina high-throughput sequencing platform; the low-quality reads containing splices were removed to ensure high-quality reads. HISAT2 (20) and StringTie (21) were used to compare transcriptome data with a reference genome sequence, and the reading distribution on the reference gene was calculated and the coverage was determined. Fragments per kilobase of transcript per million fragments mapped (FPKM) (22) was used as an index to measure transcripts or gene expression levels, and gene expression was analyzed quantitatively. Finally, three transcriptome libraries (C, D, M) were constructed.

EdgeR (23) was used for differential expression analysis of samples to obtain differential expression gene sets under variable experimental bconditions. The p-values were adjusted by applying a false discovery rate (FDR) to control false discovery rates. Genes were expressed differentially when the corrected *p*-value was < 0.05 and fold change was ≥ 1.5. The purity, concentration and integrity of RNA samples were confirmed to ensure the use of appropriate samples for transcriptome sequencing (Biomarker technologies Co. Ltd.).

### 2.7. Functional enrichment analysis

Gene Ontology (GO) and Kyoto Encyclopedia of Genes and Genomes (KEGG) pathway enrichment analysis of differentially expressed genes (DEGs) were carried out using Cytoscape (version 3.8.2) plug-in ClueGO (version 2.5.7). The KEGG pathway enrichment analysis of DEGs was implemented using R software (Version 3.4.1); KEGG pathways and GO terms were considered significantly enriched with an adjusted p-value < 0.05. ClueGO and R software was also used for clustering GO terms and visualization by Sankey diagrams.

### 2.8. Quantitative real-time PCR (qRT-pCR) for validation

Total RNA from left ventricles were extracted using Animal Total RNA Isolation Kit and the 5 × All-In-One MasterMix (with AccuRT Genomic DNA Removal kit) (ABM, China). The extraction steps of total RNA were according to the kit instructions. The EvaGreen Express 2 × qPCR MasterMix-No Dye (ABM, China) was used to prepare the qRT-PCR reaction mixture. RT-PCR was performed using a SLAN-96S Real-Time PCR system (Shanghai, China) with an amplification procedure of 95^°^C for 10 min, 40 cycles of 95^°^C for 10 s and 60^°^C for 30 s. The cycle threshold (Ct) value was used to perform calculations. The relative mRNA expression levels were calculated using the ΔCt method (where ΔCt value is obtained by subtracting the Ct value of internal reference mRNA (GAPDH) from that RNAs of selected genes). The ratios of relative expression were expressed as 2^–ΔΔ^ ^Ct^. Specific primers were designed by Sangon Biotech Co., Ltd (Chengdu, China) and presented in Table 2.

**TABLE 2:** The primers of qRT-PCR.

mRNA (GenBank no)	Primer	Size (bp)
CCN1 (83476)	Forward: GCCAGTTCCACCGCTCTGAAAG	146
	Reverse: CAGCCCACAGCACCGTCAATAC	
ERK1 (50689)	Forward: GGACCTCATGGAGACGGACCTG	90
	Reverse: CGGAGGATCTGGTAGAGGAAGTAGC	
ERK2 (116590)	Forward: TGAAGACACAGCACCTCAGCAATG	131
	Reverse: GGTGTTCAGCAGGAGGTTGGAAG	
EGR1 (24330)	Forward: GCCAGGAGTGATGAACGCAAGAG	117
	Reverse: GGATGGGTAGGAAGAGAGGGAAGAG	
GAPDH (24383)	Forward: ACAGCAACAGGGTGGTGGAC	226
	Reverse: TTTGAGGGTGCAGCGAACTT	

### 2.9. Western blotting

Total protein was extracted from the left ventricle, and processed total protein samples were separated by electrophoresis using SDS-PAGE and then transferred to PVDF membranes. Blocking incubation was performed with 5% non-fat dry milk for 1.5 h at room temperature. After washing for 30 s, the proteins were incubated within the diluted primary antibodies against CCN1, ERK1/2, EGR1, PGC-1α and GAPDH at 4°C for 24 h. After three washes with TBST buffer, the membranes were incubated with horse-radish peroxidase-conjugated secondary antibodies (Proteintech Group Inc., Wuhan, China) for 1h at room temperature. Bands were quantified by ECL luminescence reagent and analyzed by Image J. Primary antibodies against CCN1, EGR1, PGC-1α and GAPDH were purchased from HuaBio Co., Ltd. (Hangzhou, China); antibodies against ERK1/2 were purchased from Abcam (UK).

### 2.10. Citrate synthase (CS) activity assay

Left ventricular tissues were homogenized in homogenate medium and centrifuged for 10 min at 3,000-4,000 rpm and 4°C. The supernatants were prepared as 10% tissue homogenates for assay. A CS kit (Nanjing Jiancheng Bioengineering Institute, China) was used to measure CS activity levels according to the manufacturer’s instructions.

### 2.11. Statistics

Data were analyzed using SPSS26.0. The Shapiro–Wilk normality test was used to determine the normal distribution of variables and a one-way ANOVA test to compare changes between three groups. GraphPad prism 8.0.1 was used for statistical analysis. Statistically significant differences were set at *p* < 0.05. The results for indexes are presented as the mean ± standard deviation of the mean (SD).

## 3. Results

### 3.1. Type 2 diabetic rat model

20 rats in the pre-diabetic group met the criteria for type 2 diabetes, as indicated by polydipsia, polyphagia, polyuria, and blood glucose values of ≥ 16.7 mmol/L. Diabetic rats were randomly divided into group D and M of ten rats per group.

### 3.2. Blood glucose and insulin resistance

**FIGURE 1:**
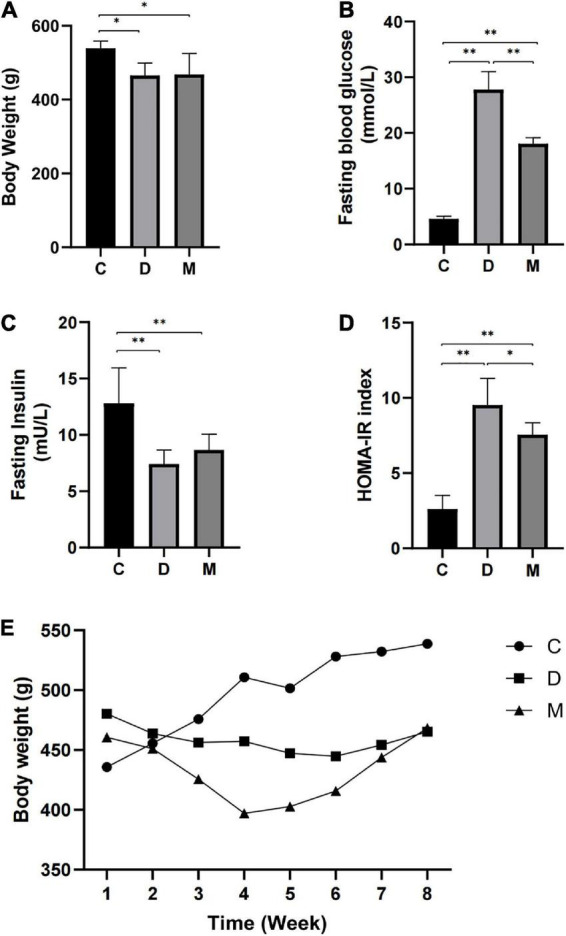
Body weight, fasting blood glucose, fasting insulin, and HOMA-IR after 8 weeks of MOTS-c treatment. **(A)** Rats in groups D and M had lower body weights compared with rats in group C (*p* < 0.01). **(B)** Rats in groups D and M had higher FBG compared with rats from group C (*p* < 0.01). Values for FBG in M were significantly lower than that in group D (*p* < 0.01). **(C)** Rats from groups D and M groups had lower FINS compared with rats from group C (*p* < 0.01). **(D)** Rats from groups D and M had higher HOMA-IR values compared with group C (*p* < 0.01). Values of HOMA-IR in group M were significantly lower than in group D (*p* < 0.05). **(E)** The weekly body weight of rats in groups C, D and M. Data are expressed as mean ± SD; *n* = 10 rats per group; *p < 0.05, ^**^p < 0.01; C, non-diabetic control group; D, diabetic control group; M, diabetic MOTS-c injection group.

Rats in the D and M groups weighed less than those in group C after 8-weeks of treatment with MOTS-c (*p* < 0.05) (Figures 1A, E). The most rapid weight loss was observed in rats with MOTS-c application, which may account for the ability of MOTS-c to increase energy metabolism (24). Fasting glucose levels were reduced in rats from group M compared with those in group D (p < 0.01), indicating that treatment with MOTS-c alleviated hyperglycemia (Figure 1B). Furthermore, insulin levels in rats from groups D and M were decreased (both *p* < 0.01) compared with those in group C, with no significant differences between groups D and M (both *p* > 0.05) (Figure 1C). The changes in fasting glucose and fasting insulin were in accordance with the results expected from the induction of type 2 diabetes by high-fat diet plus a low-dose of STZ (25).

HOMA-IR indices were calculated to assess insulin resistance. A higher HOMA-IR index was observed in rats from group D compared with rats in group C (*p* < 0.01), indicating insulin resistance. The HOMA-IR index of rats in group M were decreased in comparison with that of group D (*p* < 0.05), with significant differences between groups M and C (*p* < 0.01) (Figure 1D).

### 3.3. Cardiac structural and functional indices

**FIGURE 2:**
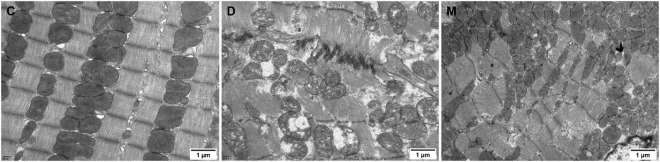
Electron micrographs of myocardium in groups C, D, M. The myocardium of rats from group C maintained continuous regular sarcomeres and constituted myofibrils, with mitochondria distributed between myofibrils that were structurally intact. The myofibrils of the myocardium in rats from group D were fragmented and disorganized, and the mitochondria were arranged in a disorganized manner with broken cristae, and with inner membranes that were swollen and appeared vacuolated or ruptured. The arrangement of myocardial sarcomeres in rats from the M group were regular, with intact mitochondrial structure with limited swelling and vacuoles.

We determined the effects of MOTS-c on diabetic myocardial ultrastructure using transmission electron microscopy. Diabetes caused cardiac myofiber disarrangement and abnormal changes of mitochondrial structure, including irregular arrangement, disrupted cristae, swelling and vacuolation of cardiac cells. Treatment of diabetic rats with MOTS-c significantly reduced myocardial mitochondrial damage, with improvements in myocardial fibers and mitochondrial structure. Moreover, we observed an obviously increase in the number of mitochondria (Figure 2).

**FIGURE 3:**
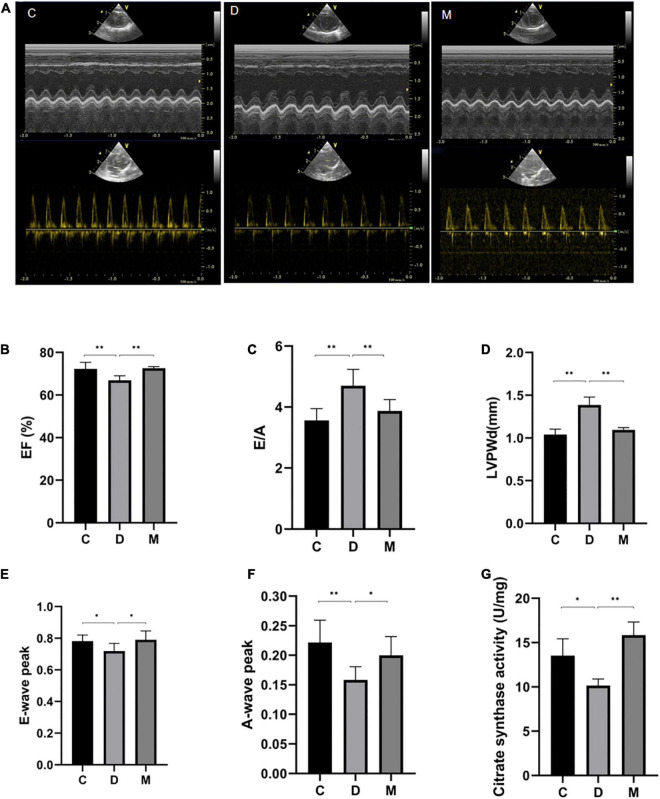
Cardiac function after 8 weeks of treatment with MOTS-c. **(A)** M-mode echocardiographic images and echocardiographic doppler color flow images from rats in groups C, D and M. **(B)** Rats from groups C and M had greater EF values compared with rats from groups D (*p* < 0.01), with no significant differences in EF between rats from groups M and C (*p* > 0.05). **(C,D)** The E/A ratio and LVPWd were increased in rats from group D compared with rats from group C (*p* < 0.01). While the E/A ratios and LVPWd in rats from group M were decreased compared with rats from group D (*p* < 0.05), there were no differences from rats in group C (*p* > 0.05). **(E)** The E-wave peak was decreased in rats in group D compared with the C group (*p* < 0.05). Rats from group M had higher E-wave peak values compared with rats from group D (*p* < 0.05), with no significant differences between rats from groups M and C (*p* > 0.05). **(F)** The A-wave peak in rats from group D was decreased compared with rats from groups C and M (*p* < 0.01, *p* < 0.05). **(G)** Citrate synthase activity in left ventricle from rats in groups C, D, and M. Data are expressed as mean ± SD; *n* = 10 rats in each group; *p < 0.05, ^**^p < 0.01; C, non-diabetic control group; D, diabetic control group; M, diabetic MOTS-c injection group.

The activity of CS was measured to determinate mitochondrial function. CS is a the rate-limiting enzyme of the tricarboxylic acid (TCA) cycle and a common biomarker of mitochondrial oxidative capacity (26). CS activity was decreased significantly in rats of group D compared with control rats (*p* < 0.05), and no difference was observed between groups C and M (*p* > 0.05) (Figure 3G), implying recovery of mitochondrial function in diabetic rats after treatment with MOTS-c.

Representative M-mode echocardiographic images from all experimental groups are shown in Figure 3. The LVPWd value of rats in group D was significantly increased, suggesting lesions in the left ventricle (*p* < 0.01) (Figure 3D). Values of EF in rats from group D were decreased compared with rats from group C (*p* < 0.01) (Figure 3B), indicating impaired cardiac systolic function of diabetic rats. We also determined that peak E-waves and A-waves of diabetic rats in group D were decreased (p < 0.05), and that the A-wave decreased more rapidly (p < 0.05), so increasing the E/A ratio (*p* < 0.05) (Figures 3C, E, F). There were no differences in EF, E/A, LVPWd, peak E-waves and A-waves from groups M and C (*p* > 0.05) (Figures 3B-F), indicating that MOTS-c targeted cardiac diastolic and systolic function.

### 3.4. Identification of differentially expressed genes

**FIGURE 4:**
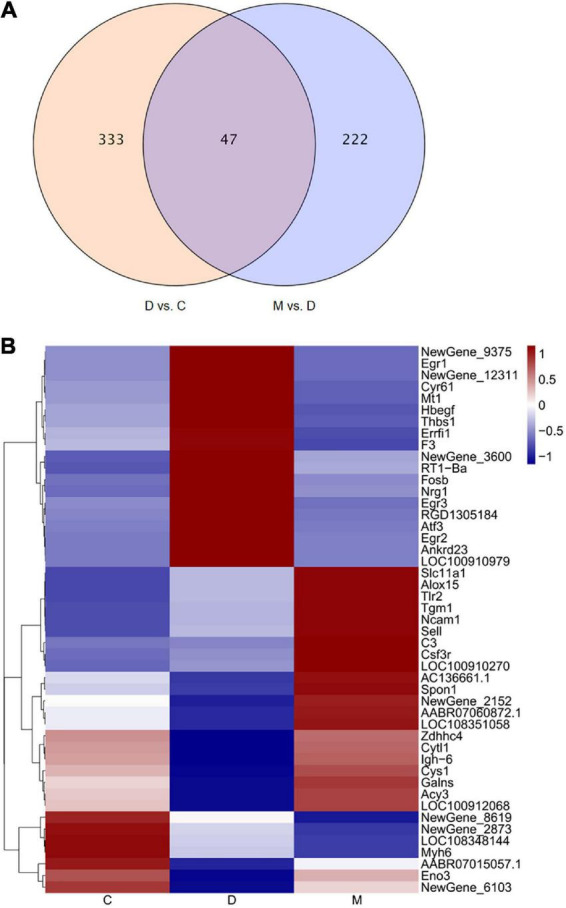
Changes in gene expression profiles. **(A)** The overlap of DEGs in the comparison D vs. C and M vs. D presented as a Venn diagram. **(B)** Heatmap representing the relative expression levels of the overlapping DEGs in the comparisons D vs. C and M vs. D. In the heatmap, red and blue represent up regulation and down regulation respectively, and white represents no change.

The transcriptome of cardiac tissues from rats in groups C, D and M of rats were profiled to explore the mechanisms involved in the adaptive responses to MOTS-c in diabetic rats. We compared cardiac tissue from D vs. C to explore pathogenic genes of diabetes, and compared M vs. D to determine DEGs in diabetic rats after 8 weeks of treatment with MOTS-c. The overlapping DEGs in the comparisons D vs. C and M vs. D were analyzed to identify the genes improved by MOTS-c as illustrated by a Venn diagram (Figure 4A). The expression patterns of the overlapping DEGs were obtained using hierarchical clustering and are presented in a heatmap (Figure 4B).

Treatment with MOTS-c for 8 weeks resulted in the differential expression of 47 genes when comparing D vs. C and M vs. D; of these, 24 genes were upregulated while 23 genes were downregulated. Among these DEGs, the gene expression of rats in group D group tended to be the opposite of rats in group C, while that of rats in groups M and C were similar.

### 3.5. Functional enrichment analysis of DEGs

**FIGURE 5:**
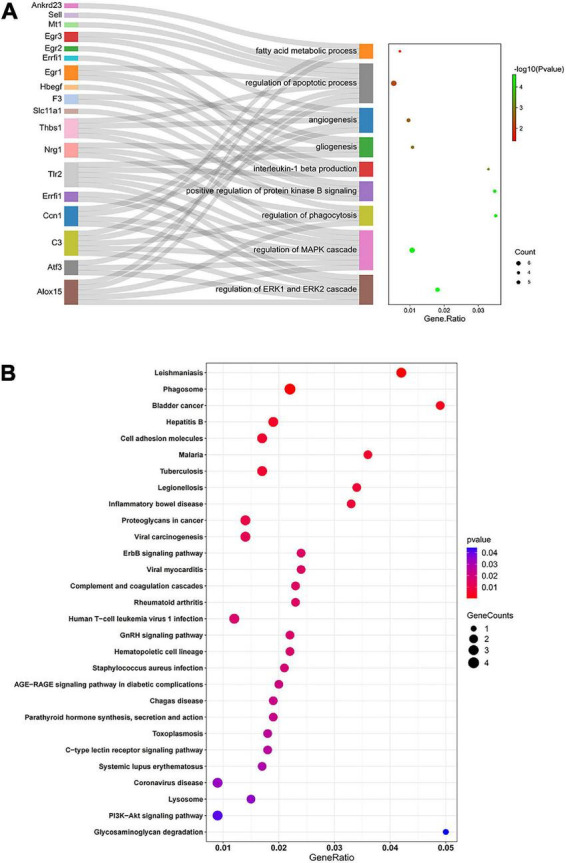
Functional enrichment analysis by GO and KEGG. **(A)** Sankey diagram based on analysis of significantly enriched GO terms of the overlapping DEGs in the comparisons D vs. C and M vs. D. DEGs were analyzed using plug-in ClueGO of Cytoscape to determine significantly enriched GO terms. Sankey diagram visualized GO terms and the genes annotated within, where only the most significant terms per group is presented. **(B)** Significantly enriched KEGG pathways of the overlapping DEGs in the comparisons D vs. C and M vs. D. In bubble chart, the color and size of the points were scaled with respect to adjusted *p*-values and the number of related DEGs. Gene ratio refers to the proportion of DEGs in each pathway.

GO describes gene products associated biological processes (BP), cellular components (CC), and molecular functions (MF). The 47 overlapping DEGs in the comparisons D vs. C and M vs. D were enriched in 195 GO terms, of which 188, 2 and 5 terms were enriched in BP, CC, and MF, respectively. After clustering, the GO terms were mostly related to angiogenesis, regulation of the apoptotic process, regulation of the MAPK cascade, positive regulation of protein kinase activity, fatty acid metabolic process, regulation of phagocytosis, positive regulation of protein kinase B signaling, regulation of ERK1/2 (extracellular regulated protein kinases) cascade, gliogenesis, and interleukin-1 beta production (Figure 5A). A KEGG pathway enrichment analysis indicated significant enrichment of 18 KEGG pathways (Figure 5B). Five signaling pathways (ErbB signaling pathway, complement and coagulation cascades, C-type lectin receptor signaling pathway, PI3K-Akt signaling pathway and AGE-RAGE signaling pathway in diabetic complications) related to immunity, apoptosis and glycometabolism were also enriched. Among the genes associated with functional annotation, ALOX15 (arachidonic acid 15-lipoxygenase-1), ATF3 (activating transcription factor 3), CCN1 (cell communication network factor 1), EGR1 (early growth response 1), EGR3 (early growth response 3), ERRFI1 (ERBB receptor feedback inhibitor 1), THBS1 (thrombospondin-1), TLR2 (toll-like receptor 2) and NRG1 (neuregulin-1) appeared at a higher frequency in the diabetic myocardium.

### 3.6. The effects of MOTS-c on CCN1/ERK1/2/EGR1 signaling pathway

#### 3.6.1. RT-PCR for validation

**FIGURE 6:**
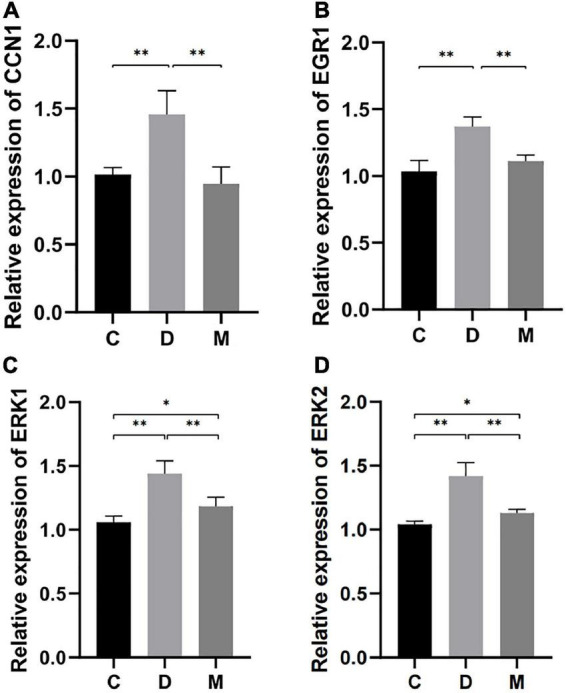
Gene expression of CCN1, EGR1, ERK1, and ERK2 in ventricular cardiomyocytes from rats in C, D, and M groups. **(A–D)** The mRNA expression of CCN1, EGR1, ERK1, and ERK2 were decreased in rats from group M compared with C and D groups (*p* < 0.01). Data are expressed as mean ± SD; *p < 0.05, ^**^p < 0.01; C, non-diabetic control group; D, diabetic control group; M, diabetic MOTS-c injection group.

Genes for CCN1 and EGR1 were highly expressed and appeared in the apoptosis related terms and CCN1 was included in the ERK1/2 cascade with the greatest enrichment. To explore the molecular mechanism by which MOTS-c improves cardiac function in diabetes, we performed transcript analysis of the highly expressed CCN1, ERK1, ERK2, and EGR1 genes by qRT-PCR. The gene expression of CCN1, ERK1/2 and EGR1 in the myocardium of diabetic rats were decreased after MOTS-c treatment (*p* < 0.01) (Figure 6).

#### 3.6.2. Western blotting for validation

**FIGURE 7:**
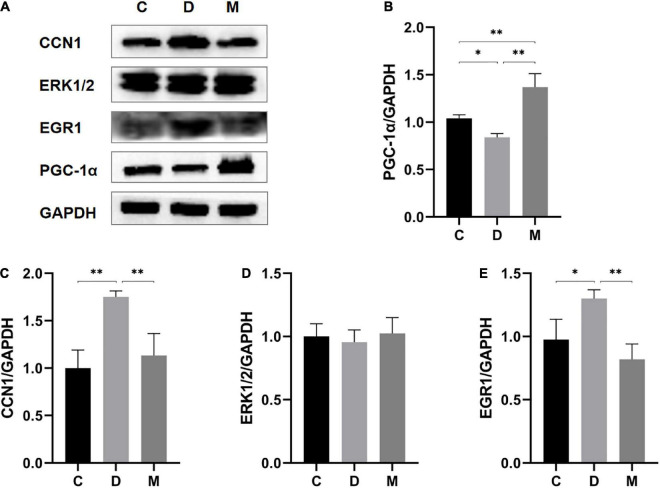
Protein expression levels of PGC-1α, CCN1, ERK1/2, and EGR1 in ventricular cardiomyocytes from rats in C, D, and M groups. **(A–E)** Western blot analysis of PGC-1α, CCN1, ERK1/2, and EGR1. Data are expressed as mean ± SD; *p < 0.05, ^**^p < 0.01; C, non-diabetic control group; D, diabetic control group; M, diabetic MOTS-c injection group.

To further support increases in mitochondrial biogenesis by MOTS-c, we measured PPARγ coactivator-1α (PGC-1α) protein (a mitochondrial biogenesis markers (27)) by western blotting. PGC-1α protein expression was significantly decreased in the myocardium of diabetic rats (p < 0.05) and increased after treatment with MOTS-c (*p* < 0.01) (Figure 7B).

To further understand the molecular details, we measured protein expression levels of CCN1 (*p* < 0.01) and EGR1 (*p* < 0.05) in diabetic rats. MOTS-c treatment could nearly restore to these changes to normal levels (all *p* < 0.01) (Figures 7C, E). However, the levels of ERK1/2 protein expression did not differ between the groups (*p* > 0.05) (Figure 7D).

## 4. Discussion

There are disturbances in calcium balance and mitochondrial function in diabetes (28, 29). Metabolic stress causes Ca^2+^ overload, leading to opening of the mitochondrial permeability transition pore and subsequent cardiomyocyte autophagy and cardiac necrosis (30). Changes in the mtDNA copy number can protect mitochondria from oxidative damage in T2D (31). The newly identified mtDNA encoded polypeptide MOTS-c improves insulin sensitivity, regulates glycolipid metabolism, and affects mitochondrial metabolism (8, 32), leading to suggestions that MOTS-c could be a new treatment for diabetes, and for reducing myocardial damage in diabetes (33).

We used a rat model of T2D to explore the effects of MOTS-c on diabetic myocardial structure and function, and used transcriptomics to better characterize the molecular mechanisms involved. Our findings show that high-fat diet combined with STZ-induced produced diabetic rats as also reported in others (34, 35). Treatment with MOTS-c for 8 weeks improved the structure and function of cardiac myofibers and mitochondria in diabetic rats. Moreover, treatment with MOTS-c increased the protein expression level of PGC-1α. PGC-1α is a master coregulator in mitochondrial biogenesis. Upon phosphorylation, PGC-1α translocates from the cytoplasm to the nucleus to trigger mitochondrial biogenesis (27). In consideration of the improved mitochondrial morphology and function in our results, it is possible that MOTS-c exerts its effects on mitochondrial biogenesis. Moreover, transcriptomic analysis indicated that ATF3 and THBS1 genes were overexpressed in diabetic rats, and decreased after MOTS-c treatment. ATF3 is a member of the ATF/cAMP response element-binding (CREB) family (36). Overexpression of ATF3 results in accumulation of depolarized mitochondria, and increased mitochondrial ROS production (37). In addition, Zhao et.al (38) reported that overexpression of THBS1 stimulated mitochondrial Ca^2+^ levels and decreased mitochondrial membrane potential (MMP) levels, leading to mitochondrial dysfunction. These data strongly suggests that MOTS-c improves mitochondrial function.

Functional enrichment analysis indicated that the main mechanisms by which MOTS-c improved diabetic heart function involved changes in fatty acid metabolism, immunoregulation, angiogenesis and apoptosis, with altered regulation of the MAPK and ERK1/2, protein kinase B and ErbB signaling pathways as indicated by functional annotation.

CCN1 and EGR1 genes, as GO terms related to apotosis, were highly expressed in transcriptomics analysis. CCN1 was annotated in the ERK1/2 cascade pathway with the highest enrichment; ERK1/2 alleviates cardiomyocyte apoptosis by regulating EGR1 (39). Hence, we propose that CCN1/ERK1/2/EGR1 is important for reducing myocardial injury in diabetes, as supported by our findings with RT-PCR and western blotting analysis indicating that MOTS-c reduced mRNA expression levels of CCN1, ERK1/2 and EGR1, and protein expression levels of CCN1 and EGR1 in the hearts of diabetic rats, but without affecting ERK1/2 total protein expression (Figures 6, 7).

Levels of the matricellular protein CCN1 are increased in the cardiomyocytes of a stressed heart to promote cardiomyocyte apoptosis (40). CCN1 is overexpressed in the myocardium of diabetic rats, which can stimulate autophagy and decrease myocardial function (41). Although there were no reports on the targeting of CCN1, AMPK, some studies indicate reduced expression of CCN1 by its ectopically expressed siRNA (42). It is possible that AMPK links MOTS-c to CCN1. CCN1 triggers ROS accumulation and mitochondrial outer membrane permeabilization (MOMP) by binding to integrin α6β1 and activation of ERK and JNK to target ubiquitinated mitochondria and stimulate autophagy (43). MOTS-c enhanced mitochondrial homeostasis by decreasing ROS production (44), and CCN1 inhibition may play an important role in this process.

CCN1 also increases levels of the myocardial apoptotic molecule Fas ligand (FasL), which activates the ERK1/2 pathway, leading to dilated cardiomyopathy and advanced heart failure (43, 45, 46). ERKs are a widely conserved family of serine/threonine protein kinases that are implicated in many cellular programs such as cell proliferation, differentiation and apoptosis. MOTS-c participates in various physiological activities by regulating levels of activated ERK1/2 (47, 48). Some studies indicate that MOTS-c administration was without effect on total ERK1/2 protein expression (47, 48), which is consistent with our data. Activation of ERK1/2 mediates cardiac hypertrophy and dysfunction, and in addition to its anti-apoptotic effects in the myocardium (49, 50). The ERK/EGR1 signaling pathway has a critical role in the process of apoptosis in multiple organs (39, 51, 52). Inhibition of ERK1/2 kinase expression downregulates EGR1 expression, thereby reducing myocardial ischemia-reperfusion induced apoptosis and autophagy (39). Our findings suggest that MOTS-c reduces myocardial apoptosis and repairs myocardial mitochondrial injury by inhibiting the overexpression of CCN1 and the downstream activation of ERK1/2, and decreasing the expression of EGR1.

The expression of the tricarboxylic acid (TCA) cycle substrate–dependent mitochondrial calcium uptake 1(MICU1) protein is mediated by the transcription factor EGR1 (53). MICU1 is up-regulated during nutrient deficiency and EGR1 is required for nutrient stress-induced MICU1 upregulation for control of mitochondrial Ca^2+^ uptake (53). Decreased cardiomyocyte function is in part mediated by abnormal mitochondrial calcium handling and a decreased levels of free matrix calcium levels (54), implying that MOTS-c most likely regulates mitochondrial calcium ion homeostasis imbalance by inhibiting EGR1 expression.

### 4.1. Study limitations

Our study has some limitations: (1) We only measured coding RNA (mRNA) levels and did not investigate the role of non-coding RNA in MOTS-c induced improvements in diabetic heart disease by MOTS-c; (2) We did not investigate the transcriptomic profiling characters of the effects of different MOTS-c treatment protocols such as: different concentration, route and frequency of administration on the diabetic myocardium.

## 5. Conclusion

**FIGURE 8:**
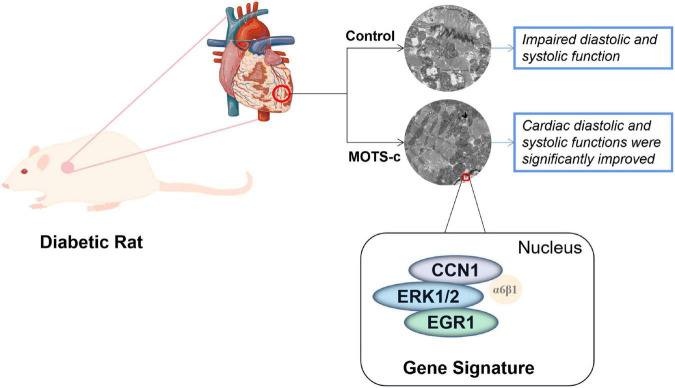
A conceptual model of MOTS-c improving myocardial injury in diabetic rats by inhibiting CCN1/ERK/EGR1 expression. Change: Cardiac diastolic and systolic functions were significantly improved to: Improved cardiac diastolic and systolic function.

An eight-week treatment with MOTS-c treatment reduced cardiac dysfunction in diabetes, which was related to changes in 47 genes involved in fatty acid metabolism, immunoregulation, angiogenesis and apoptosis. Signaling by CCN1/ERK1/2/EGR1 may play an important role in MOTS-c inhibiting myocardial apoptosis. Thus, MOTS-c not only inhibits the development of diabetes and attenuates diabetic myocardial lesions by multiple pathways, including attenuation of mitophagy, reducing mitochondrial damage, and inhibiting myocardial apoptosis through the CCN1/ERK/EGR1 pathway, as illustrated in Figure 8. Our findings provide the first experimental evidence of the molecular mechanisms of improvements in cardiac function after treatment of diabetic rats with MOTS-c, and indicate new strategies in the prevention and treatment of diabetic cardiac dysfunction in diabetes.

## Data availability statement

The original contributions presented in this study are included in the article/Supplementary material, further inquiries can be directed to the corresponding author.

## Ethics statement

This animal study was reviewed and approved by experiments were approved by the Academic Committee of Chengdu Sport Institute (No.: 2021-07).

## Author contributions

MW and SL: conceptualization. MW: methodology, writing—original draft preparation, and visualization. MW, XP, and JM: software. XP, YP, and JY: validation. YF: formal analysis. MW and JM: data curation. GW, SL, and IL: writing—review and editing. SL: supervision, project administration, and funding acquisition. All authors read and agreed to the published version of the manuscript.
